# 6-Bromo-1,3-dimethyl-1*H*-imidazo[4,5-*b*]pyridin-2(3*H*)-one

**DOI:** 10.1107/S1600536810025134

**Published:** 2010-07-03

**Authors:** Siham Dahmani, Youssef Kandri Rodi, Hafid Zouihri, El Mokhtar Essassi, Seik Weng Ng

**Affiliations:** a Laboratoire de Chimie Organique Appliquée, Faculté des Sciences et Techniques, Université Sidi Mohamed Ben Abdallah, Fès, Morocco; b CNRST Division UATRS, Angle Allal Fassi/FAR, BP 8027 Hay Riad, Rabat, Morocco; c Laboratoire de Chimie Organique Hétérocyclique, Pôle de Compétences Pharmacochimie, Université Mohammed V-Agdal, BP 1014 Avenue Ibn Batout, Rabat, Morocco; d Department of Chemistry, University of Malaya, 50603 Kuala Lumpur, Malaysia

## Abstract

The non-H atoms of the two independent mol­ecules in the asymmetric unit of the title compound, C_8_H_8_BrN_3_O, are planar (r.m.s. deviations = 0.015 and 0.019 Å). In the crystal, the mol­ecules are linked into a zigzag chain along the *c* axis by C—H⋯O hydrogen bonds.

## Related literature

For the synthesis of the un-brominated compound, see: Yutilov *et al.* (1998 ▶, 2005 ▶).
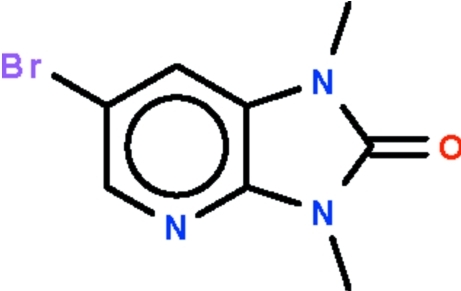


## Experimental

### 

#### Crystal data

C_8_H_8_BrN_3_O
*M* *_r_* = 242.08
Monoclinic, 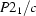

*a* = 21.7981 (4) Å
*b* = 3.9929 (1) Å
*c* = 20.6636 (3) Å
β = 95.398 (1)°
*V* = 1790.53 (6) Å^3^
*Z* = 8
Mo *K*α radiation
μ = 4.55 mm^−1^
*T* = 293 K
0.25 × 0.20 × 0.15 mm

#### Data collection

Bruker X8 APEXII area-detector diffractometer
Absorption correction: multi-scan (*SADABS*; Sheldrick, 1996 ▶) *T*_min_ = 0.396, *T*_max_ = 0.548
21586 measured reflections
5229 independent reflections
3539 reflections with *I* > 2σ(*I*)
*R*_int_ = 0.036

#### Refinement

*R*[*F* ^2^ > 2σ(*F* ^2^)] = 0.031
*wR*(*F* ^2^) = 0.086
*S* = 1.01
5229 reflections
239 parameters
H-atom parameters constrained
Δρ_max_ = 0.28 e Å^−3^
Δρ_min_ = −0.29 e Å^−3^

### 

Data collection: *APEX2* (Bruker, 2008 ▶); cell refinement: *SAINT* (Bruker, 2008 ▶); data reduction: *SAINT*; program(s) used to solve structure: *SHELXS97* (Sheldrick, 2008 ▶); program(s) used to refine structure: *SHELXL97* (Sheldrick, 2008 ▶); molecular graphics: *X-SEED* (Barbour, 2001 ▶); software used to prepare material for publication: *publCIF* (Westrip, 2010 ▶).

## Supplementary Material

Crystal structure: contains datablocks global, I. DOI: 10.1107/S1600536810025134/ci5119sup1.cif

Structure factors: contains datablocks I. DOI: 10.1107/S1600536810025134/ci5119Isup2.hkl

Additional supplementary materials:  crystallographic information; 3D view; checkCIF report

## Figures and Tables

**Table 1:** Hydrogen-bond geometry (Å, °)

*D*—H⋯*A*	*D*—H	H⋯*A*	*D*⋯*A*	*D*—H⋯*A*
C8—H8*C*⋯O2^i^	0.96	2.33	3.279 (3)	167
C11—H11⋯O1	0.93	2.49	3.321 (3)	148

Symmetry code: (i) 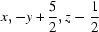.

**Fig. 1.**
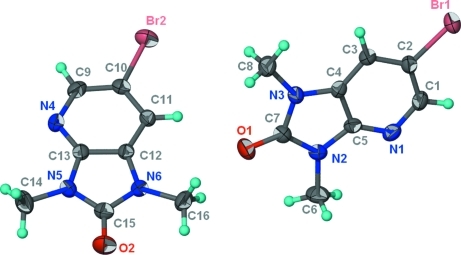
Displacement ellipsoid plot (Barbour, 2001) of the two independent molecules of C8H8BrN3O at the 50% probability level. H atoms are shown as spheres of arbitrary radii.

## supplementary crystallographic information

### Comment

The imidazo[4,5-*b*]pyridine unit is an important heterocyclic nucleus found in a large number of medicinal compounds. The synthesis of 1,3-dimethyl-1,3-dihydro-imidazo[4,5-*b*]pyridin-2-one involves several steps (Yutilov *et al.*, 1998, 2005). The 6-bromo derivative (Scheme I) is conveniently synthesized by the direct reaction of 6-bromo-1,3-dihydro-imidazo[4,5-*b*]pyridin-2-one with methyl iodide.

The aysmmetric unit (Fig. 1) has two independent molecules. Both are planar.

### Experimental

To a mixture of 6-bromo-1,3-dihydro-imidazo[4,5-*b*]pyridin-2-one (0.15 g, 1 mmol), potassium carbonate (0.38 g, 4 mmol) and tetra-*n*-butylammonium bromide (0.02 g, 0.1 mmol) in DMF (10 ml) was added methyl iodide (0.1 ml, 2.5 mmol). Stirring was continued at room temperature for 12 h. After the completion of reaction (as monitored by TLC), the mixture was filtered and the solvent removed under reduced pressure. The residue was purified by column chromatography on silica gel with ethyl acetate-hexane (1:3) as eluent. The compound was recrystallized from ethyl acetate-hexane (1:3) to afford colourless crystals.

### Refinement

H atoms were placed in calculated positions (C-H = 0.93–0.96 Å) and were included in the refinement in the riding model approximation, with *U*(H) set to 1.2–1.5*U*_eq_(C).

### Crystal data

**Table:**

C_8_H_8_BrN_3_O	*F*(000) = 960
*M**_r_* = 242.08	*D*_x_ = 1.796 Mg m^−^^3^
Monoclinic, *P*2_1_/*c*	Mo *K*α radiation, λ = 0.71073 Å
Hall symbol: -P 2ybc	Cell parameters from 6375 reflections
*a* = 21.7981 (4) Å	θ = 2.6–25.2°
*b* = 3.9929 (1) Å	µ = 4.55 mm^−^^1^
*c* = 20.6636 (3) Å	*T* = 293 K
β = 95.398 (1)°	Prism, colourless
*V* = 1790.53 (6) Å^3^	0.25 × 0.20 × 0.15 mm
*Z* = 8	

### Data collection

**Table:**

Bruker X8 APEXII area-detector diffractometer	5229 independent reflections
Radiation source: fine-focus sealed tube	3539 reflections with *I* > 2σ(*I*)
graphite	*R*_int_ = 0.036
φ and ω scans	θ_max_ = 30.0°, θ_min_ = 2.6°
Absorption correction: multi-scan (*SADABS*; Sheldrick, 1996)	*h* = −30→28
*T*_min_ = 0.396, *T*_max_ = 0.548	*k* = −5→5
21586 measured reflections	*l* = −29→28

### Refinement

**Table:**

Refinement on *F*^2^	Primary atom site location: structure-invariant direct methods
Least-squares matrix: full	Secondary atom site location: difference Fourier map
*R*[*F*^2^ > 2σ(*F*^2^)] = 0.031	Hydrogen site location: inferred from neighbouring sites
*wR*(*F*^2^) = 0.086	H-atom parameters constrained
*S* = 1.01	*w* = 1/[σ^2^(*F*_o_^2^) + (0.0438*P*)^2^ + 0.1144*P*] where *P* = (*F*_o_^2^ + 2*F*_c_^2^)/3
5229 reflections	(Δ/σ)_max_ = 0.001
239 parameters	Δρ_max_ = 0.28 e Å^−^^3^
0 restraints	Δρ_min_ = −0.29 e Å^−^^3^

### Fractional atomic coordinates and isotropic or equivalent isotropic displacement parameters (Å^2^)

**Table:**

	*x*	*y*	*z*	*U*_iso_*/*U*_eq_	
Br1	0.407800 (11)	0.91753 (7)	0.468246 (11)	0.05023 (9)	
N1	0.44555 (8)	0.5071 (5)	0.65032 (9)	0.0416 (4)	
N2	0.37942 (8)	0.4903 (5)	0.73723 (8)	0.0400 (4)	
N3	0.29799 (8)	0.7480 (5)	0.68786 (8)	0.0387 (4)	
N4	0.05385 (8)	0.5642 (5)	0.92033 (9)	0.0400 (4)	
N5	0.12070 (8)	0.8692 (5)	0.99889 (8)	0.0391 (4)	
N6	0.19961 (8)	0.9449 (5)	0.94033 (8)	0.0373 (4)	
O1	0.29345 (8)	0.5668 (5)	0.79455 (8)	0.0568 (5)	
O2	0.20597 (8)	1.1650 (5)	1.04514 (8)	0.0576 (5)	
C1	0.44708 (10)	0.6147 (6)	0.58839 (11)	0.0421 (5)	
H1	0.4826	0.5767	0.5678	0.050*	
C2	0.39881 (10)	0.7769 (6)	0.55465 (10)	0.0374 (5)	
C3	0.34349 (10)	0.8436 (5)	0.58128 (10)	0.0356 (5)	
H3	0.3105	0.9528	0.5585	0.043*	
C4	0.34205 (9)	0.7335 (5)	0.64419 (10)	0.0339 (4)	
C5	0.39361 (9)	0.5704 (5)	0.67562 (10)	0.0345 (5)	
C6	0.42023 (12)	0.3314 (7)	0.78847 (13)	0.0546 (7)	
H6A	0.3960	0.2231	0.8188	0.082*	
H6B	0.4460	0.4983	0.8107	0.082*	
H6C	0.4455	0.1684	0.7696	0.082*	
C7	0.32072 (10)	0.5976 (6)	0.74587 (11)	0.0411 (5)	
C8	0.23694 (10)	0.8923 (7)	0.67783 (13)	0.0503 (6)	
H8A	0.2289	1.0214	0.7153	0.075*	
H8B	0.2070	0.7164	0.6713	0.075*	
H8C	0.2344	1.0348	0.6402	0.075*	
C9	0.05044 (10)	0.4500 (6)	0.85870 (11)	0.0415 (5)	
H9	0.0149	0.3385	0.8423	0.050*	
C10	0.09723 (10)	0.4905 (5)	0.81873 (10)	0.0356 (5)	
C11	0.15197 (9)	0.6529 (5)	0.83943 (10)	0.0350 (5)	
H11	0.1839	0.6795	0.8130	0.042*	
C12	0.15516 (9)	0.7711 (5)	0.90210 (9)	0.0311 (4)	
C13	0.10513 (9)	0.7226 (5)	0.93943 (10)	0.0337 (4)	
C14	0.08264 (13)	0.8834 (8)	1.05313 (13)	0.0621 (8)	
H14A	0.1083	0.8604	1.0933	0.093*	
H14B	0.0530	0.7048	1.0492	0.093*	
H14C	0.0615	1.0945	1.0526	0.093*	
C15	0.17830 (10)	1.0112 (6)	0.99985 (11)	0.0408 (5)	
C16	0.25863 (10)	1.0616 (6)	0.92138 (12)	0.0474 (6)	
H16A	0.2842	1.1318	0.9593	0.071*	
H16B	0.2520	1.2468	0.8919	0.071*	
H16C	0.2786	0.8829	0.9004	0.071*	
Br2	0.086179 (12)	0.32023 (7)	0.732957 (11)	0.05183 (9)	

### Atomic displacement parameters (Å^2^)

**Table:**

	*U*^11^	*U*^22^	*U*^33^	*U*^12^	*U*^13^	*U*^23^
Br1	0.05040 (16)	0.06094 (17)	0.04140 (14)	−0.00344 (12)	0.01514 (11)	0.00544 (11)
N1	0.0294 (9)	0.0492 (12)	0.0453 (11)	0.0036 (8)	−0.0008 (8)	−0.0047 (8)
N2	0.0366 (10)	0.0488 (12)	0.0335 (10)	0.0022 (8)	−0.0025 (8)	0.0032 (8)
N3	0.0332 (10)	0.0493 (11)	0.0343 (9)	0.0062 (8)	0.0063 (7)	0.0052 (8)
N4	0.0318 (10)	0.0484 (11)	0.0403 (10)	−0.0036 (8)	0.0061 (8)	0.0035 (8)
N5	0.0396 (10)	0.0478 (11)	0.0306 (9)	0.0006 (8)	0.0071 (8)	−0.0029 (8)
N6	0.0332 (9)	0.0425 (10)	0.0357 (9)	−0.0038 (8)	0.0012 (7)	0.0002 (8)
O1	0.0547 (10)	0.0782 (13)	0.0395 (9)	0.0077 (9)	0.0152 (8)	0.0125 (8)
O2	0.0627 (12)	0.0644 (12)	0.0439 (10)	−0.0092 (9)	−0.0042 (9)	−0.0157 (8)
C1	0.0303 (11)	0.0507 (14)	0.0463 (13)	−0.0013 (10)	0.0096 (10)	−0.0058 (10)
C2	0.0362 (12)	0.0429 (12)	0.0337 (11)	−0.0065 (10)	0.0069 (9)	−0.0025 (9)
C3	0.0322 (11)	0.0394 (12)	0.0350 (11)	0.0000 (9)	0.0015 (9)	0.0010 (8)
C4	0.0289 (11)	0.0356 (11)	0.0373 (11)	−0.0003 (9)	0.0045 (8)	−0.0016 (9)
C5	0.0332 (11)	0.0370 (11)	0.0322 (11)	−0.0027 (9)	−0.0028 (8)	−0.0038 (8)
C6	0.0565 (16)	0.0608 (17)	0.0441 (13)	0.0095 (13)	−0.0084 (11)	0.0098 (12)
C7	0.0400 (12)	0.0477 (13)	0.0357 (12)	0.0000 (10)	0.0041 (9)	0.0006 (9)
C8	0.0385 (13)	0.0615 (16)	0.0520 (14)	0.0104 (11)	0.0101 (11)	0.0106 (12)
C9	0.0333 (12)	0.0433 (13)	0.0463 (13)	−0.0043 (10)	−0.0046 (10)	0.0021 (10)
C10	0.0405 (12)	0.0358 (12)	0.0295 (10)	0.0029 (9)	−0.0013 (9)	0.0024 (8)
C11	0.0338 (11)	0.0393 (12)	0.0321 (11)	0.0031 (9)	0.0048 (8)	0.0051 (8)
C12	0.0293 (10)	0.0320 (11)	0.0318 (10)	0.0012 (8)	0.0013 (8)	0.0039 (8)
C13	0.0321 (11)	0.0362 (11)	0.0327 (10)	0.0040 (9)	0.0028 (8)	0.0046 (8)
C14	0.0624 (17)	0.082 (2)	0.0457 (14)	−0.0051 (15)	0.0220 (13)	−0.0141 (13)
C15	0.0435 (13)	0.0423 (13)	0.0357 (11)	0.0036 (10)	−0.0010 (10)	0.0011 (9)
C16	0.0336 (12)	0.0531 (14)	0.0557 (15)	−0.0068 (11)	0.0049 (10)	0.0010 (11)
Br2	0.06391 (18)	0.05326 (16)	0.03660 (13)	−0.00056 (12)	−0.00434 (11)	−0.00705 (10)

### Geometric parameters (Å, °)

**Table:**

Br1—C2	1.899 (2)	C3—C4	1.375 (3)
N1—C5	1.316 (3)	C3—H3	0.93
N1—C1	1.353 (3)	C4—C5	1.404 (3)
N2—C5	1.376 (3)	C6—H6A	0.96
N2—C7	1.377 (3)	C6—H6B	0.96
N2—C6	1.462 (3)	C6—H6C	0.96
N3—C4	1.380 (3)	C8—H8A	0.96
N3—C7	1.390 (3)	C8—H8B	0.96
N3—C8	1.447 (3)	C8—H8C	0.96
N4—C13	1.313 (3)	C9—C10	1.381 (3)
N4—C9	1.348 (3)	C9—H9	0.93
N5—C13	1.374 (3)	C10—C11	1.390 (3)
N5—C15	1.376 (3)	C10—Br2	1.893 (2)
N5—C14	1.457 (3)	C11—C12	1.374 (3)
N6—C12	1.379 (3)	C11—H11	0.93
N6—C15	1.381 (3)	C12—C13	1.407 (3)
N6—C16	1.456 (3)	C14—H14A	0.96
O1—C7	1.222 (3)	C14—H14B	0.96
O2—C15	1.229 (3)	C14—H14C	0.96
C1—C2	1.369 (3)	C16—H16A	0.96
C1—H1	0.93	C16—H16B	0.96
C2—C3	1.397 (3)	C16—H16C	0.96
			
C5—N1—C1	114.43 (18)	N2—C7—N3	106.43 (18)
C5—N2—C7	109.90 (17)	N3—C8—H8A	109.5
C5—N2—C6	126.63 (19)	N3—C8—H8B	109.5
C7—N2—C6	123.40 (19)	H8A—C8—H8B	109.5
C4—N3—C7	109.41 (18)	N3—C8—H8C	109.5
C4—N3—C8	127.62 (19)	H8A—C8—H8C	109.5
C7—N3—C8	122.96 (19)	H8B—C8—H8C	109.5
C13—N4—C9	114.63 (18)	N4—C9—C10	123.2 (2)
C13—N5—C15	109.67 (18)	N4—C9—H9	118.4
C13—N5—C14	126.4 (2)	C10—C9—H9	118.4
C15—N5—C14	123.9 (2)	C9—C10—C11	122.1 (2)
C12—N6—C15	109.26 (17)	C9—C10—Br2	118.81 (16)
C12—N6—C16	126.73 (18)	C11—C10—Br2	119.06 (16)
C15—N6—C16	123.92 (19)	C12—C11—C10	114.59 (19)
N1—C1—C2	123.1 (2)	C12—C11—H11	122.7
N1—C1—H1	118.5	C10—C11—H11	122.7
C2—C1—H1	118.5	N6—C12—C11	133.1 (2)
C1—C2—C3	122.7 (2)	N6—C12—C13	107.11 (17)
C1—C2—Br1	118.44 (16)	C11—C12—C13	119.8 (2)
C3—C2—Br1	118.88 (16)	N4—C13—N5	127.28 (19)
C4—C3—C2	114.1 (2)	N4—C13—C12	125.70 (19)
C4—C3—H3	122.9	N5—C13—C12	107.02 (18)
C2—C3—H3	122.9	N5—C14—H14A	109.5
C3—C4—N3	133.2 (2)	N5—C14—H14B	109.5
C3—C4—C5	119.79 (19)	H14A—C14—H14B	109.5
N3—C4—C5	107.04 (18)	N5—C14—H14C	109.5
N1—C5—N2	126.9 (2)	H14A—C14—H14C	109.5
N1—C5—C4	125.9 (2)	H14B—C14—H14C	109.5
N2—C5—C4	107.22 (18)	O2—C15—N6	126.3 (2)
N2—C6—H6A	109.5	O2—C15—N5	126.8 (2)
N2—C6—H6B	109.5	N6—C15—N5	106.92 (18)
H6A—C6—H6B	109.5	N6—C16—H16A	109.5
N2—C6—H6C	109.5	N6—C16—H16B	109.5
H6A—C6—H6C	109.5	H16A—C16—H16B	109.5
H6B—C6—H6C	109.5	N6—C16—H16C	109.5
O1—C7—N2	127.3 (2)	H16A—C16—H16C	109.5
O1—C7—N3	126.3 (2)	H16B—C16—H16C	109.5
			
C5—N1—C1—C2	0.2 (3)	C13—N4—C9—C10	0.9 (3)
N1—C1—C2—C3	−0.4 (4)	N4—C9—C10—C11	0.2 (3)
N1—C1—C2—Br1	179.39 (17)	N4—C9—C10—Br2	−179.59 (17)
C1—C2—C3—C4	0.3 (3)	C9—C10—C11—C12	−0.6 (3)
Br1—C2—C3—C4	−179.52 (15)	Br2—C10—C11—C12	179.19 (15)
C2—C3—C4—N3	179.6 (2)	C15—N6—C12—C11	178.1 (2)
C2—C3—C4—C5	0.0 (3)	C16—N6—C12—C11	1.5 (4)
C7—N3—C4—C3	−179.7 (2)	C15—N6—C12—C13	−1.1 (2)
C8—N3—C4—C3	0.5 (4)	C16—N6—C12—C13	−177.7 (2)
C7—N3—C4—C5	−0.1 (2)	C10—C11—C12—N6	−179.2 (2)
C8—N3—C4—C5	−179.9 (2)	C10—C11—C12—C13	−0.1 (3)
C1—N1—C5—N2	−179.9 (2)	C9—N4—C13—N5	178.9 (2)
C1—N1—C5—C4	0.1 (3)	C9—N4—C13—C12	−1.7 (3)
C7—N2—C5—N1	180.0 (2)	C15—N5—C13—N4	−179.7 (2)
C6—N2—C5—N1	3.0 (4)	C14—N5—C13—N4	−1.1 (4)
C7—N2—C5—C4	−0.1 (2)	C15—N5—C13—C12	0.8 (2)
C6—N2—C5—C4	−177.1 (2)	C14—N5—C13—C12	179.4 (2)
C3—C4—C5—N1	−0.3 (3)	N6—C12—C13—N4	−179.3 (2)
N3—C4—C5—N1	−180.0 (2)	C11—C12—C13—N4	1.4 (3)
C3—C4—C5—N2	179.80 (19)	N6—C12—C13—N5	0.2 (2)
N3—C4—C5—N2	0.1 (2)	C11—C12—C13—N5	−179.13 (19)
C5—N2—C7—O1	179.8 (2)	C12—N6—C15—O2	−179.2 (2)
C6—N2—C7—O1	−3.1 (4)	C16—N6—C15—O2	−2.4 (4)
C5—N2—C7—N3	0.1 (3)	C12—N6—C15—N5	1.5 (2)
C6—N2—C7—N3	177.2 (2)	C16—N6—C15—N5	178.30 (19)
C4—N3—C7—O1	−179.7 (2)	C13—N5—C15—O2	179.3 (2)
C8—N3—C7—O1	0.1 (4)	C14—N5—C15—O2	0.6 (4)
C4—N3—C7—N2	0.0 (3)	C13—N5—C15—N6	−1.4 (2)
C8—N3—C7—N2	179.8 (2)	C14—N5—C15—N6	179.9 (2)

### Hydrogen-bond geometry (Å, °)

**Table:**

*D*—H···*A*	*D*—H	H···*A*	*D*···*A*	*D*—H···*A*
C8—H8C···O2^i^	0.96	2.33	3.279 (3)	167
C11—H11···O1	0.93	2.49	3.321 (3)	148

Symmetry codes: (i) *x*, −*y*+5/2, *z*−1/2.
